# Atrial Flow Regulator for Postcapillary Pulmonary Hypertension: First-in-Human Transcatheter AFR Device Implantations in RCM

**DOI:** 10.1016/j.jaccas.2022.05.010

**Published:** 2022-05-31

**Authors:** Georg Hansmann, Anna Sabiniewicz, Robert Sabiniewicz

**Affiliations:** a Department of Pediatric Cardiology and Critical Care, Hannover Medical School, Hannover, Germany; b European Pediatric Pulmonary Vascular Disease Network, Berlin, Germany; c Department of Pediatric Cardiology and Congenital Heart Disease, Medical University of Gdańsk, Gdańsk, Poland

**Keywords:** atrial flow regulator device, heart failure, left atrial hypertension, pulmonary hypertension, pulmonary vascular disease, restrictive cardiomyopathy, transcatheter intervention, AFR, atrial flow regulator, HF, heart failure, HFpEF, heart failure with preserved ejection fraction, HTx, heart transplantation, LA, left atrium, LAP, left atrial pressure, LV, left ventricle, LVEDP, left ventricular end-diastolic pressure, NYHA, New York Heart Association, MR, magnetic resonance, PAWP, pulmonary artery wedge pressure, PH, pulmonary hypertension, PVR, pulmonary vascular resistance, RCM, restrictive cardiomyopathy

## Abstract

Restrictive cardiomyopathy (RCM) has a poor prognosis and limited treatment options apart from heart transplantation (HTx). We report on the first-in-human interventional atrial flow regulator (AFR) implantations in 3 children with RCM, leading to marked clinical and hemodynamic improvement. We propose the AFR as bridge to HTx or destination therapy in RCM. (**Level of Difficulty: Advanced.**)

## Central Illustration

**Figure:**
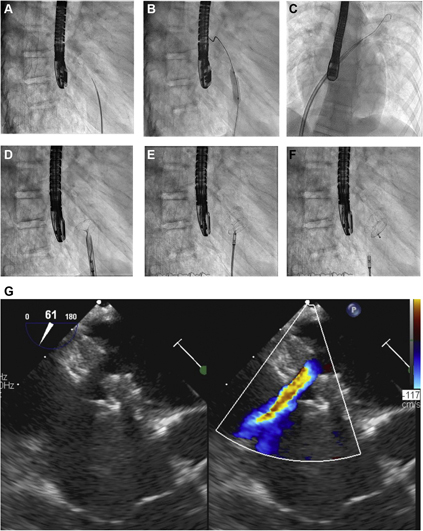

Patients with left heart failure (HF) regularly experience exercise-induced dyspnea that is caused by elevated left atrial pressure (LAP) secondary to severe diastolic dysfunction and increased filling (end-diastolic) pressures of the left ventricle (LV). The consequent left atrial (LA) hypertension causes pulmonary congestion and postcapillary pulmonary hypertension (PH), which is associated with high mortality and morbidity.^1^ Increased LV end-diastolic pressures (LVEDP) are observed in HF patients with both reduced (HFrEF) and preserved (HFpEF) LV ejection fraction. Treatment options are limited mainly to diuretics, mineralocorticoid receptor antagonists, and lifestyle changes. Importantly, isolated postcapillary PH (Ipc-PH) in HFpEF patients can progress to combined pre- and postcapillary PH,^2^, ^3^, ^4^ and ultimately to right ventricular pressure overload and failure.

**Learning Objectives**

• To understand that creating an interatrial communication with an AFR device improves LA volume overload, postcapillary PH, and HF symptoms (treatment goals).
• To recognize that early AFR device implantation can be a bridge to HTx or a destination therapy in RCM.

Device-based solutions to improve cardiac physiology in HFpEF and to prevent the aforementioned isolated postcapillary PH and its progression combined pre- and postcapillary PH have been proposed:^5^ The atrial flow regulator (AFR) (Occlutech) is a self-expandable double-disc nitinol wire mesh construction allowing communication across the interatrial septum. The AFRs with fenestration diameters of 8 or 10 mm for HF patients have the European CE mark. The U.S. Food and Drug Administration (FDA) granted conditional approval of an Investigational Device Exemption for the AFR to treat heart failure in 2021.

Although the AFR device has been implanted in older adults (>60 years of age) with HFpEF/HFrEF and LA hypertension,^6^ and younger adults with severe pulmonary arterial hypertension presenting with syncope and right ventricular failure,^7^^,^^8^ to date, there is no published report on AFR implantation in any children with HFpEF/HFrEF. Moreover, to the best of our knowledge, there is no published report on AFR implantation for the indication of restrictive cardiomyopathy (RCM) in either children or adults.

## Patient 1

A 12-year-old girl first noticed dyspnea on exertion and general weakness, followed by progressive fatigue and fading exercise tolerance, to the extent that she could barely walk (New York Heart Association [NYHA] HF functional class III). Echocardiography enabled the diagnosis of LV-predominant cardiomyopathy with LA enlargement.

Cardiac magnetic resonance (MR) on admission showed a severely enlarged LA (end-systolic volume 77 mL/m^2^) and a nondilated, nonhypertrophied LV (LVEDV 77 mL/m^2^) with preserved systolic function (LVEF 58%). There was no late gadolinium enhancement, no clear evidence of myocardial edema, and no signs of noncompaction of the LV. Subsequent cardiac catheterization, 1 year after the first symptoms revealed low right-sided and very high left-sided filling pressures, mildly elevated mean pulmonary artery pressure (mPAP 25 mm Hg; mPAP/mSAP = 0.44) and diastolic pulmonary artery pressure (dPAP 16 mm Hg), a pulmonary vascular resistance (PVR) index <3 WU/m^2^, and normal cardiac index, qualifying her as having HFpEF with mild PH (Table 1).

**Table 1:** Clinical and Hemodynamic Characteristics of 3 Children With Restrictive Cardiomyopathy Before and After AFR Device Implantation

	Patient 1		Patient 2		Patient 3	
	Time 1 (Before AFR)	Time 2 (After AFR)	Time 1 (Before AFR)	Time 2 (After AFR)	Time 1 (Before AFR)	Time 2 (After AFR)
Demographics						
Age (y)	13	13-14	12	12	6	6
Male or female	F	F	F	F	F	F
Height (m)	1.60	1.63	1.46	1.46	1.00	1.00
Weight (kg)	36.0	40.8	58	58	13.9	13.9
Body surface area (m^2^)	1.26	1.36	1.50	1.05	0.62	0.62
Diagnosis						
Cardiomyopathy (age at diagnosis)	RCM (12 y)	RCM	RCM (9 y)	RCM	RCM (2 y)	RCM
PH group	Group 2 PH	Group 2 PH	Group 2 PH	Group 2 PH	Group 2 PH	Group 2 PH
Comorbidities	None	None	Developmental retardation	Developmental retardation	None	None
NYHA functional class	3	2	3-4	3-4	3	2
Biomarker						
Date	Time 1	3 mo later	Time 1	1 mo later	Time 1	1 mo later
NTproBNP (pg/mL)	1,232	342	3,054	2,870	7,162	5,352
Medication	Eplerenone	Eplerenone	Carvedilol, spironolactone, furosemide	Carvedilol, spironolactone, furosemide	Lisinopril, spironolactone, furosemide	Lisinopril, spironolactone, furosemide
Hemodynamics (catheterization)						
Date	Time 1 (before AFR)	8 mo later	Time 1	10 min later	Time 1	
mRAP (mm Hg)	1	5	15	20	26	
RVEDP (mm Hg)	5	11	11	12	10	
sPAP (mm Hg)	47	32	53	44	54	
**mPAP (mm Hg)**	**30**	**19 (−37%)**	**40**	**31 (−22%)**	**46**	
**dPAP (mm Hg)**	**18**	**11 (−39%)**	**33**	**24 (−27%)**	**39**	
sSAP/mSAP/dPAP (mm Hg)	75/60/51	76/59/50	87/58/43	73/46/32	93/77/69	
mPAP/mSAP	0.5	0.32 (−36%)	0.68	0.67	0.59	
**PAWP (mm Hg)**	**20**	**13 (−35%)**	**29**	**21 (−28%)**	**37**	
LVEDP (mm Hg)	23	18 (−22%)	−	−	23	
**mTPG (mm Hg)**	**10**	**6 (−40%)**	**29**	**25 (−14%)**	**9**	
dTPG (mm Hg)	−2	−2	14	11 (−21%)	2	
PVRi (WU/m^2^)	2.36	1.19 (−50%)	3.23	2.56	3.20	
PVR/SVR	0.17	0.14	0.26	0.29	0.16	
Qpi	4.23	7.74	3.40	3.9 (+15%)	2.8	
Qsi (= cardiac index)	4.23	5.95	3.40	3.0 (−12%)	2.6	
**Qp/Qs**	**1.0**	**1.30 (+30%)**	**1.0**	**1.31 (+31%)**	**1.07**	
Cardiac MR imaging						
Date	Time 1	10 mo later (2 wk after AFR)			Time 1	1 mo later (4 wk after AFR)
RV mass index (g/m^2^)	21	18				
RAESV index (mL/m^2^)	−	−			17	16
RVEDV index (mL/m^2^)	54	68			58	62
RVES index (mL/m^2^)	10	16			26	35
RVSV index (mL/m^2^)	44	52			32	27
RVEF (%)	81	76			56	43
LV mass index (g/m^2^)	53	45			46	34
**LAESV index (mL/m^2^)**	**77**	**37 (−52%)**			**28**	**18 (−36%)**
LVEDV index (mL/m^2^)	77	73			54	63
LVESV index (mL/m^2^)	32	22			28	30
LVSV index (mL/m^2^)	45	22			25	33
**LVEF (%)**	**58**	**70 (+21%)**			**47**	**53 (+13%)**
RVEDV/LVEDV ratio	0.70	0.93			1.07	0.98
RVESV/LVESV ratio	0.31	0.72			0.92	1.16

Patient 1 and 2 received an AFR device with 8-mm diameter fenestration. Patient 3 received an AFR with 6 mm diameter fenestration. The follow-up studies included cardiac magnetic resonance imaging and/or cardiac catheterization, as indicated, and serial echocardiograms (not shown). All 3 patients improved clinically and hemodynamically after (s/p) AFR device implantation. Patient 1 (Hannover) underwent genetic testing that revealed a heterozygous mutation in the FLNC gene, encoding for filamin C, an actin-cross-linking protein that is expressed in heart and skeletal muscle and endomyocardial biopsies at a second cardiac catheterization several months before AFR device implantation (see main text). Patient 2 (Gdansk) was not a heart transplant candidate (psychomotor developmental retardation), detoriated and ultimately died 25 months after AFR device implantation. Patient 3 (Gdansk) was diagnosed at the age of 2 years with RCM, underwent genetic testing that detected a c.559 deletion in the TNNI3 gene, encoding for troponin I3, the inhibitory subunit of the troponin complex. Mutations in the TNNI3 gene are known to cause familial hypertrophic cardiomyopathy type 7 (CMH7) and familial restrictive cardiomyopathy (RCM). Patient 3 underwent AFR-implantation at the age of 6 years. Patient 1 and 3 are alive as of April 2022, and improved to NYHA heart failure functional class 2. Patient 3 is actively listed for heart transplantation. The variables that changed the most with AFR implantation (effect size) are in **bold** font.

AFR = atrial flow regulator; dPAP = diastolic pulmonary artery pressure; dSAP = diastolic systemic arterial pressure; dTPG = diastolic transpulmonary pressure gradient; LAESV = left atrial end-systolic volume; LV = left ventricle; LVEDP = left ventricular end-diastolic pressure; LVEDV = left ventricular end-diastolic volume; LVEF = left ventricular ejection fraction; LVESV = left ventricular end-systolic volume; LVSV = left ventricular stroke volume; mPAP = mean pulmonary artery pressure; MR = magnetic resonance; mRAP = mean right atrial pressure; mSAP = mean systemic arterial pressure; mTPG = mean transpulmonary pressure gradient; NTproBNP = N-terminal pro-brain natriuretic peptide; PAWP = pulmonary artery wedge pressure; PVRi = pulmonary vascular resistance index; Qpi = pulmonary blood flow index; Qsi = systemic blood flow index; RAESV = right atrial end-systolic volume; RV = right ventricle; RVEDP = right ventricular end-diastolic pressure; RVEDV = right ventricular end-diastolic volume; RVEF = right ventricular ejection fraction; RVESV = right ventricular end-systolic volume; RVESV = right ventricular end-systolic volume; RVSV = right ventricular stroke volume; sPAP = systolic pulmonary artery pressure; sSAP = systolic systemic artery pressure.

Endomyocardial biopsy and histologic analysis demonstrated cardiac fibrosis, cardiac texture anomalies, and microangiopathy, but no signs of myocarditis, consistent with the cardiac MR study. Genetic testing revealed a heterozygous mutation in the *FLNC* gene, encoding for filamin C, leading to the diagnosis of RCM with myofibrillar myopathy. Eplerenone monotherapy was started.

**Figure 1:**
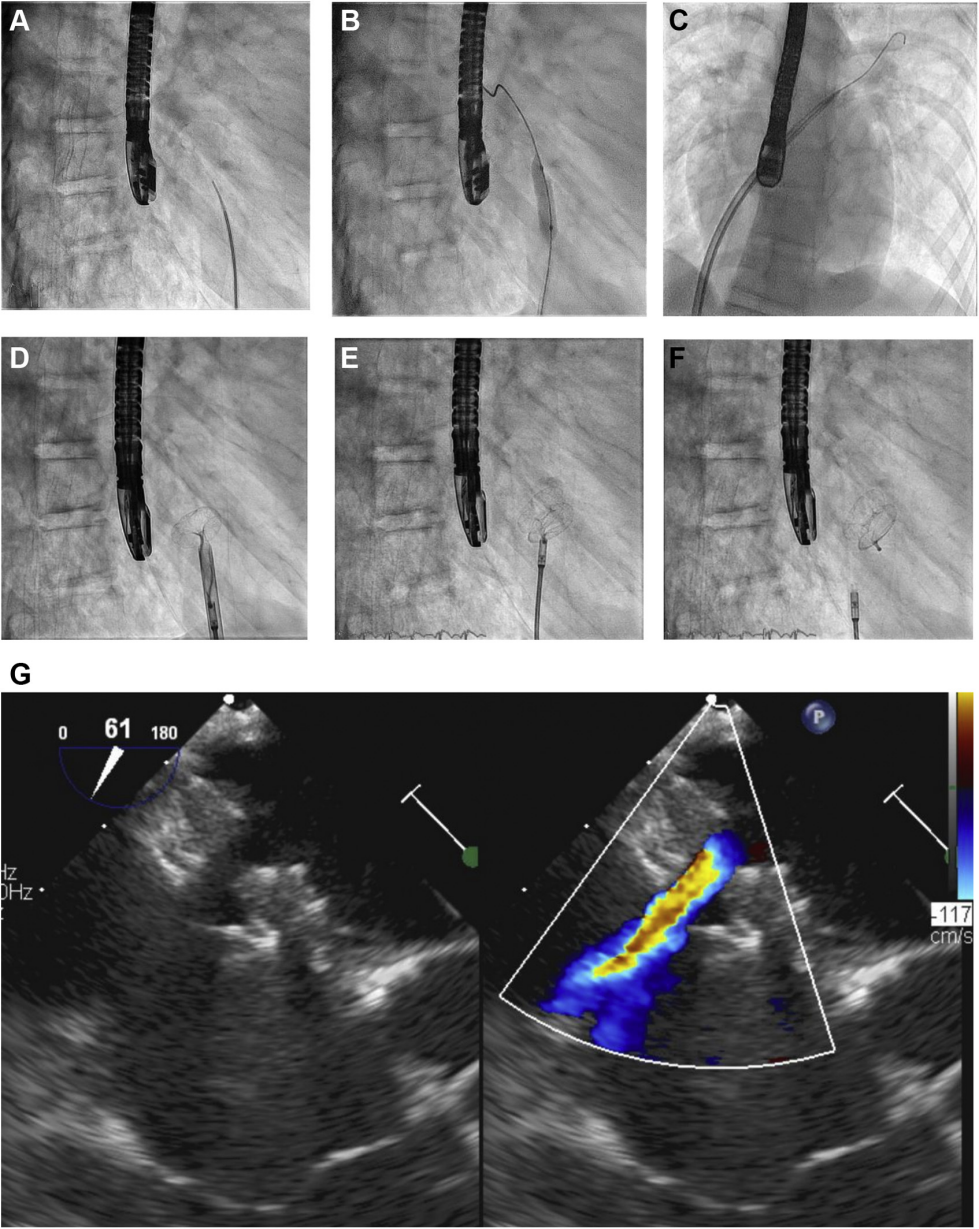
Interventional Percutaneous Implantation of the Atrial Flow Regulator-Device in a 13-Year-Old Girl With Restrictive Cardiomyopathy, Heart Failure With Preserved Ejection Fraction, and Mild Pulmonary Hypertension **(A)** Atrial transseptal puncture with Brockenbrough needle. **(B)** Balloon dilation of the atrial septum (Cordis Powerflex, 8 mm × 3 cm). **(C)** A guide wire has been positioned in the left upper pulmonary vein and a 12-F long delivery sheath is advanced across the atrial septum. **(D)** Deployment of the left atrial disc of the 8-mm atrial flow regulator (AFR) device. **(E)** The right atrial disc is deployed, and the AFR device is properly positioned. **(F)** After a pull maneuver, the AFR device has been released. **(G)** Transesophageal echocardiogram shows good position of the AFR device and restrictive atrial left-to-right shunt (6 mm).

**Figure 2:**
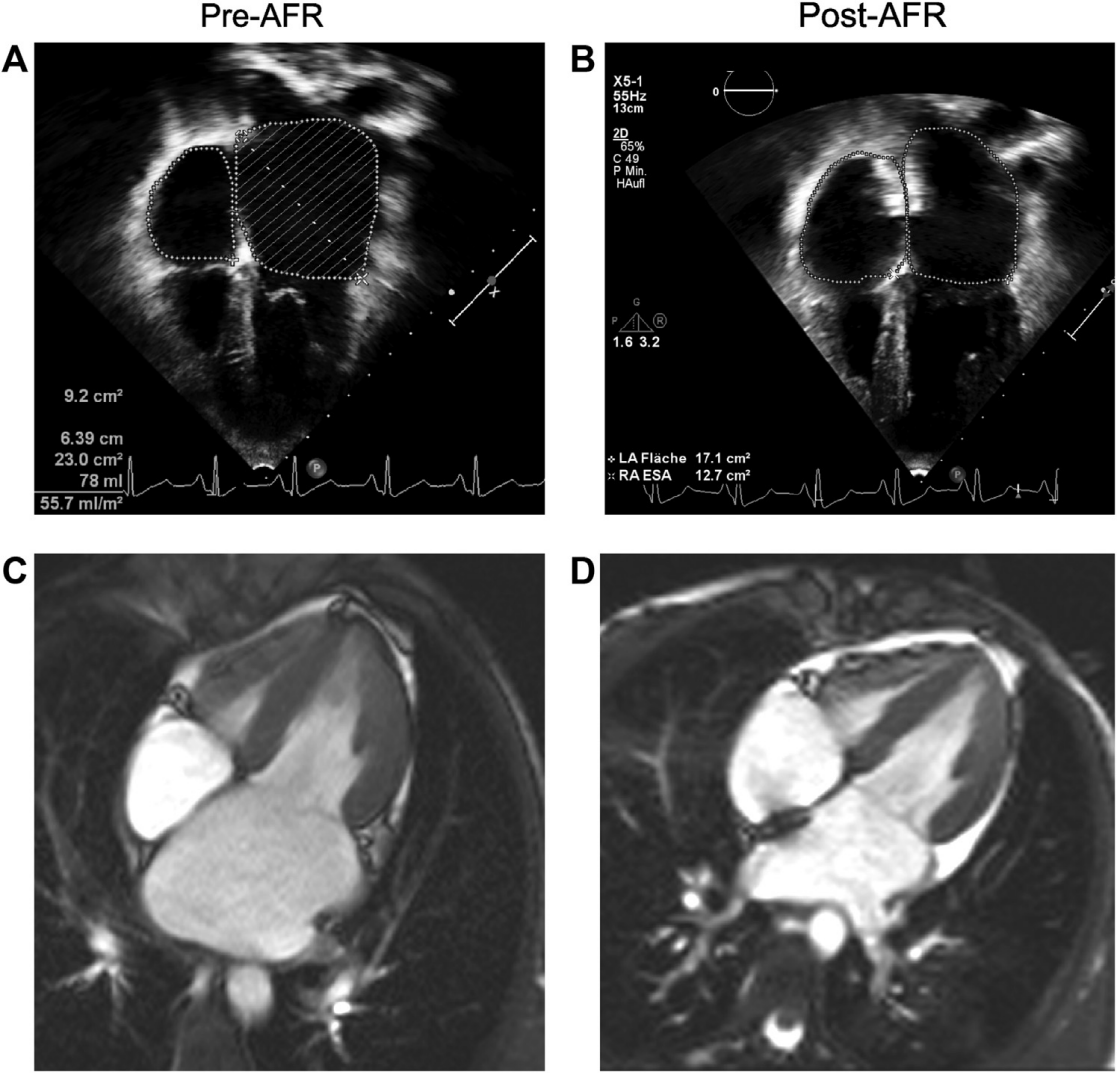
Implantation of the Atrial Flow Regulator Device Gradually Decreases Pulmonary Venous and Left Atrial Size, Indicating Decompression of the Left Atrium in a 13-Year-Old Girl With Restrictive Cardiomyopathy **(A, B)** Transthoracic echocardiogram demonstrates grossly enlarged pulmonary veins and left atrium before **(A)** and 2 weeks after **(B)** implantation of the atrial flow regulator (AFR) device. The AFR device is in adequate position. The end-systolic left atrial (LA) area has decreased from 23 cm^2^**(A)** to 17.1 cm^2^**(B)**. **(C, D)** Cardiac magnetic resonance 2 weeks after AFR device implantation shows a strong decrease in LA size, a moderate increase in right atrial (RA) size, and the AFR device in adequate position. End-systolic cine still frames are shown.

Later, 20 to 22 months after the first symptoms, the 13-year-old teenager underwent repeated cardiac catheterization for hemodynamic assessment and AFR device implantation (Figure 1, Videos 1, 2, 3, and 4). Before the procedure, the echocardiographic LVEF was normal (63%). Invasive hemodynamic data indicated moderate progression of PH (mPAP 30 mm Hg, pulmonary artery wedge pressure [PAWP] 20 mm Hg, mPAP/mSAP 0.5, dPAP 18 mm Hg, LVEDP 23 mm Hg, PVR index 2.36 WU/m^2^) (Table 1). Subsequently, an AFR device (8-mm fenestration) was successfully implanted without any complications (Figures 1 and 2, Videos 1, 2, 3, 4, 5, 6, 7, and 8, Supplemental Appendix). The size of the dilated LA and pulmonary veins slowly decreased over the following weeks, and the continuous-wave Doppler mean gradient across the AFR device decreased from 14 (pre-AFR) to 7 mm Hg in the 2 weeks after AFR implantation. At that time point, cardiac MR showed that the left atrial end-systolic volume index had decreased from 77 mL/m^2^ to 37 mL/m^2^ (−52%) and pulmonary blood flow index/systemic blood flow index had increased from 0.94 to 1.52 after the AFR implantation (Figure 2, Table 1), indicating sufficient pressure unloading of the LA and restrictive but significant left-to-right interatrial shunting (Figure 1G). Already 2 weeks after the procedure, she reported increased appetite, weight gain (+1.5 kg), and improved mood and exercise tolerance (NYHA HF functional class II). Eight months after AFR implantation, the patient walked 728 meters in 6 minutes and underwent follow-up cardiac catheterization, which demonstrated normal mPAP (19 mm Hg), normal PVR index (1.19 WU/m^2^), and improved left-sided filling pressures (PAWP 13 mm Hg, mLAP 14 mm Hg, LVEDP 18 mm Hg, Qp/Qs 1.3) (Table 1).

## Patient 2

A 9-year-old girl with a diagnosis of RCM in severe heart failure (NYHA HF functional class III-IV) required frequent hospitalizations to intensify pharmacotherapy (Table 1). At the time of her first cardiac catheterization, the echocardiographic LVEF was 50%. An AFR device was successfully implanted 2 years after the diagnosis of RCM. The procedure reduced her PAWP from 29 mm Hg to 21 mm Hg immediately after AFR implantation, and subsequently, the patient improved clinically, with decreased edema/ascites. She was not a candidate for transplantation because of psychomotor developmental retardation. Her condition deteriorated, and she ultimately died 25 months after AFR device implantation.

## Patient 3

A 6-year-old girl received a diagnosis of RCM when she was 2 years old. Genetic testing uncovered a deletion in the *TNNI3* gene, encoding for troponin I3. She had gradual deterioration in hemodynamics and experienced a few incidents of deterioration/decompensation despite pharmacotherapy (NYHA HF functional class III), so that she was listed for heart transplantation (HTx) at the age of 4 years. Cardiac catheterization with AFR implantation (6-mm fenestration) was performed: Subsequently, her clinical condition, NTproBNP, and LA size (left atrial end-systolic volume −36% by cardiac MR) improved within 1 month of follow-up care (Table 1).

## Discussion

### Restrictive cardiomyopathy

The cause of RCM is diverse and includes infiltrative conditions, storage diseases, noninfiltrative conditions (including myofibrillar myopathies and sarcomeric protein disorders), and endomyocardial processes (eg, endocardial fibroelastosis, anthracycline-induced). The prognosis of familial and nonfamilial RCM is poor and is even worse in children than in adults: According to the Pediatric Cardiomyopathy Registry, the 1-year and 5-year transplantation-free survival rates for pure RCM in children are only 48% and 22%, respectively.^9^ Interventional reduction of elevated LA pressure is an important treatment goal in RCM.

### Previous studies on AFR implantation in adults with HFpEF or HFrEF

A meta-analysis of 6 studies on a total of 226 patients explored the feasibility and efficacy of transcatheter interatrial shunt devices for chronic heart failure (3 different devices).^10^ The authors concluded that the implantation of these devices in patients with chronic HF is feasible and is associated with improved submaximal exercise capacity, improved health-related quality of life, and reduction in PAWP.^10^

The prospective, nonrandomized, multicenter phase 2 PRELIEVE study (Pilot Study to Assess Safety and Efficacy of a Novel Atrial Flow Regulator in Heart Failure Patients) reported the first-in-human use of the AFR for older patients with HFpEF (LVEF ≥40%; n = 24) or HFrEF (LVEF 15%-39%; n = 29). The resting PAWP decreased by 5 mm Hg (median) at 3 months after the AFR implantation.^6^ No shunt occlusion, stroke, or new right HF was observed during the 1-year follow-up period, with clinical improvements in certain patients.^6^

Here, we report the first-in-human transcatheter AFR device implantations in RCM. The transcatheter procedure was feasible and safe in 3 children, 6 to 13 years old, and it improved LA dilation, postcapillary pulmonary hypertension, and HF symptoms. The AFR creates permanent interatrial communication, improves quality of life, and likely extends survival in HFpEF/HfrEF; however, these findings should be confirmed in future prospective studies in patients with cardiomyopathy and LA hypertension.

## Conclusions

Creating an interatrial communication is a therapeutic option for patients with HFpEF and HFrEF, including RCM. Early AFR device implantation can be considered a bridge to HTx in young patients with RCM and a destination therapy in those who are not HTx candidates.

## Funding Support and Author Disclosures

This report was funded by a grant from the German Research Foundation (DFG KFO311, HA4348/6-2 to Dr Hansmann). Dr. Hansmann has received funding from the Federal Ministry of Education and Research (BMBF; 01KC2001B, 03VP08053) and the European Pediatric Pulmonary Vascular Disease Network (www.pvdnetwork.org). Dr. Sabiniewicz is a consultant for Occlutech, the producer of the atrial flow regulator device. All other authors have reported that they have no relationships relevant to the contents of this paper to disclose.

## Appendix

Supplemental Video 1 Fluoroscopy, AFR device delivery, device still connected

Supplemental Video 2 Fluoroscopy, AFR device delivery, device release

Supplemental Video 3 Transesophageal echocardiogram, AFR device delivery

Supplemental Video 4 Transesophageal echocardiogram (color compare), AFR device released

Supplemental Video 5 Transthoracic echocardiogram, 4CV, before AFR device implantation

Supplemental Video 6 Transthoracic echocardiogram, 4CV, after AFR device implantation

Supplemental Video 7 Cardiac MR (cine), 4CV, before AFR device implantation

Supplemental Video 8 Cardiac MR (cine), 4CV, after AFR device implantation
