# Supplementary material for: Atrial Flow Regulator for Postcapillary Pulmonary Hypertension: First-in-Human Transcatheter AFR Device Implantations in RCM
Source: JACC Case Rep. 2022 May 31;4(14):878–84. doi: 10.1016/j.jaccas.2022.05.010 (PMC9334142; doi:10.1016/j.jaccas.2022.05.010)
Supplement: Supplemental Data [file mmc9.docx]

**SUPPLEMENTAL APPENDIX**

**Transcatheter Implantation of the Atrial Flow Regulator (AFR, Occlutech)**

The technical aspects of AFR implantation have been described elsewhere[^1^](#_ENREF_1). Briefly, the interventional procedure consists of the following steps (**Figure 1**):

1. Sizing according to pulmonary arterial wedge pressure (PAWP) and septal thickness (in pediatrics, in most cases, the following AFR device is chosen: diameter D1=8mm, H=5mm, atrial discs D2=21mm, requiring a 12F femoral venous sheath).

2. Atrial transseptal puncture (Brockenbrough needle, **Figure 1A**).

3. Balloon atrial septostomy, i.e., here balloon dilation of the atrial septum (**Figure 2B**). For an 8mm AFR device, balloon inner diameters of 8-12mm are chosen. The balloon size determines the magnitude of the postprocedural, restrictive left-to-right atrial shunt.

5. Positioning of a guide wire (Cook 260cm, 0.035 inch, THSF 35x260x3) in the left upper pulmonary vein.

5. AFR device positioning and deployment of the left and right atrial disc (**Figure 2D,E**)

6. Push/Pull maneuver

7. Release of the AFR device (**Figure 2F**)

**Introduction**

Device-based solutions to improve cardiac physiology in HFpEF have been proposed[^2^](#_ENREF_2). Three different transcatheter atrial shunt devices are available for patients with either HFpEF or HFrEF: The interatrial shunt device (IASD, Corvia Medical Inc., Tewksbury, MA, USA)[^3-5^](#_ENREF_3), the V-Wave shunt (V-Wave Ltd, Caesarea, Israel)[^6^](#_ENREF_6), the Atrial Flow Regulator (AFR, Occlutech, Heslingborg, Sweden)[^1^](#_ENREF_1). The Atrial Flow Regulator (AFR) is a self-expandable double-disc nitinol wire mesh construction allowing communication across the interatrial septum. The device is fully repositionable and retrievable. The offered fenestration diameter ranges from 4 mm to 10 mm, but for HF patients, only the 8 mm and 10 mm AFRs have the European CE mark. The Food and Drug Administration (FDA) granted conditional approval of an Investigational Device Exemption (IDE) for the AFR to treat heart failure in 2021.

While the AFR device has been implanted in older adults (> 60 years old) with HFpEF/HFrEF and LA hypertension (PRELIEVE, a non-randomized phase 2 study)[^1^](#_ENREF_1), as well as younger adults with severe PAH presenting with syncope and RV failure (case series)[^7^](#_ENREF_7)^,^ [^8^](#_ENREF_8), to date, there is no report on AFR implantations in any children with HFpEF/HFrEF published. Moreover, to the best of our knowledge, there has been no published report on AFR-implantation for the indication “restrictive cardiomyopathy” (RCM), neither in children nor in adults.

***Restrictive Cardiomyopathy (RCM)***

The etiology of RCM is diverse, and includes infiltrative conditions (amyloidosis, sarcoidosis, primary hyperoxaluria – all rare causes in children), storage diseases, non-infiltrative causes (including myofibrillar myopathies and sarcomeric protein disorders) and endomyocardial processes (e.g., endocardial fibroelastosis, anthracycline-induced)[^9^](#_ENREF_9). The defining patterns of RCM are: one or both atria enlarged relative to ventricles of normal or small size with evidence of impaired diastolic filling, in the absence of significant valvular heart disease[^10^](#_ENREF_10). Prognosis of familial and non-familial RCM is poor, and even worse in children vs. adults[^11^](#_ENREF_11): According to the Pediatric Cardiomyopathy Registry (PCMR), the 1- and 5-year transplantation-free survival for pure RCM in children is only 48 and 22%, respectively[^12^](#_ENREF_12). Other small, retrospective studies reported for children a mean survival of 2 years from the time of diagnosis “RCM”[^13^](#_ENREF_13).

Interventional reduction of elevated LA pressure is an important treatment goal in RCM to prevent the development of relevant postcapillary PH (Ipc-PH), or even combined pre- and postcapillary PH (Cpc-PH); Cpc-PH is characterized by more than mild PVR-elevation (≥ 3 WU in adults and ≥ 3 WU x m^2^ in children), in addition to high left-sided filling pressures. In most transplant centers, a PVR > 8 WU (> 8 WU x m^2^ in children) at baseline - and certainly any such values with acute vasoreadibility testing - would preclude heart transplantation (HTx), and require heart and lung transplantation in these young patients.

**Case Description (Cases #1, #2, #3)**

***Case #1***

A 12 year old female noticed dyspnea on exertion and general weakness, when she had protracted, incomplete recovery from atypical pneumonia. During the COVID-19 lockdown, she stopped any sports, had progressive fatigue and fading cardiopulmonary exercise tolerance. Her heart failure symptoms were severe, to the extend that she could barely walk (NYHA HF class 3). Based on the subsequent outpatient echocardiogram, the diagnosis of a LV-predominant cardiomyopathy with LA enlargement was made.

(…)

Following the AFR-implantation, the patient reported clinical improvement by means of increased appetite, weight gain (+1.5 kg), and all, improved mood, general activity and cardiopulmonary exercise tolerance (NYHA HF class decreased from 3 to 2).

**Case #2**

A 9 year old female was diagnosed with RCM in severe heart failure (NYHA HF class 3-4: peripheral edema, hepatomegaly, ascites, dyspnea, fading exercise tolerance, fatigue). Oral medication included carvedilol, spironolactone and furosemide. The AFR-implantation immediately reduced the pulmonary arterial wedge pressure (PAWP) from 29 to 21mmHg, and subsequently, the patient improved clinically (decreased peripheral edema and ascites, reduction in diuretics). The patient was not a transplant candidate for several reasons, deteriorated and ultimately died 25 months after AFR-device implantation.

**Case #3**

A 6 year old girl was diagnosed at 2 years of age with RCM. Genetic testing uncovered a deletion in the TNNI3 gene, endcoding for troponin I3. Several cardiac catheterizations and echocardiograms documented gradual deterioration in hemodynamics. Few incidents of clinical deterioration and decompensation occurred despite pharmacotherapy (lisinopril, spironolactone, furosemide), so that she was listed for heart transplantation (HTx) at the age of 4 years. After the most recent cardiac catheterization with hemodynamic evaluation and subsequent AFR implantation (6mm fenestration), clinical condition (NYHA FC), HF biomarker (NTproBNP), and LA size (LAESV -36% by cardiac MRI) improved within 1 month of follow-up (**Table 1**).

**Discussion**

***Restrictive Cardiomyopathy (RCM)***

The etiology of RCM is diverse, and includes infiltrative conditions (amyloidosis, sarcoidosis, primary hyperoxaluria – all rare causes in children), storage diseases, non-infiltrative causes (including myofibrillar myopathies and sarcomeric protein disorders) and endomyocardial processes (e.g., endocardial fibroelastosis, anthracycline-induced)[^9^](#_ENREF_9). Prognosis of familial and non-familial RCM is poor, and even worse in children vs. adults: According to the Pediatric Cardiomyopathy Registry (PCMR), the 1- and 5-year transplantation-free survival for pure RCM in children is only 48 and 22%, respectively[^12^](#_ENREF_12). Interventional reduction of elevated LA pressure is an important treatment goal in RCM to prevent the development of relevant postcapillary PH (Ipc-PH).

***Previous Studies on AFR-Implantation in adults with HFpEF or HFrEF***

A meta-analysis of 6 studies on a total of 226 patients explored the feasibility and efficacy of transcatheter interatrial shunt devices (IASD) for chronic heart failure (3 different devices: Corvia IASD II, v-wave system/1^st^ and 2^nd^ generation, Occlutech AFR)[^14^](#_ENREF_14). The authors concluded that IASD implantation in chronic HF is feasible and associates with improved submaximal exercise capacity (measured by 6-minute walk distance), health-related quality of life, and reductions in PAWP[^14^](#_ENREF_14).

The very recent, prospective, non-randomized, multi-center PRELIEVE study[^1^](#_ENREF_1) reported the first-in-human use of the AFR (Occlutech) for older patients suffering from heart failure with preserved (HFpEF, defined in that study as LVEF ≥ 40%; n=24) or reduced (HFrEF; LVEF 15-39%; n=29) LV ejection fraction (median age 67-71 years, NYHA class III or ambulatory class IV). Of note, the 2021 ESC heart failure guidelines define now HFrEF as LVEF ≤ 40%, HF with mildly reduced EF (HFmrEF) as LVEF 41-49%, and HFpEF as LVEF ≥ 50% plus elevated LVEDP and natriuretic peptides[^15^](#_ENREF_15). In the PRELIEVE feasibility study on HFpEF/HFrEF, the resting PAWP decreased by 5 (−12) mmHg (p=0.0003, median Q1, Q3) at 3 months after the AFR implantation[^1^](#_ENREF_1). No shunt occlusion, stroke or new right HF was observed during the 1-year follow-up, with clinical improvements in certain patients[^1^](#_ENREF_1). The ongoing PROLONGER study[^16^](#_ENREF_16) investigates the clinical and hemodynamic variables that define adult HFpEF patients who may particularly benefit from AFR implantation.

Here, we report the first-in-human Atrial Flow Regulator (AFR) device implantations in restrictive cardiomyopathy. The procedure was feasible and safe in three children (6-12 years old), and improved LA dilation, postcapillary pulmonary hypertension, and heart failure symptoms in the setting of severe LV diastolic dysfunction and consecutive LA hypertension. AFR implantation creates permanent inter-atrial communication. In all previous publications, long term device patency is reported[^1^](#_ENREF_1)^,^ [^2^](#_ENREF_2). Thus, AFR implantation is a new therapeutic option for patients with restrictive cardiomyopathy. The AFR improves quality of life and likely extends the survival, increasing the chance of a successful heart transplantation. Based on our preliminary experience, we suggest that early AFR-device implantation can be considered as bridge to HTx in young patients with RCM, and as destination therapy in those who are not HTx candidates.

**Figure 1.** *Interventional, percutaneous implantation of the Atrial Flow Regulator-Device in a 13 year old female with restrictive cardiomyopathy, heart failure with preserved ejection fraction (HFpEF), and mild pulmonary hypertension (PH).* **A**, atrial transseptal puncture (Brockenbrough needle). **B**, balloon atrial septostomy, i.e., here balloon dilation of the atrial septum. For an 8mm AFR device, balloon inner diameters of 8-12mm are chosen; here: Cordis Powerflex balloon, 8mm x3 cm). **C**, a guide wire (Cook 0.035 inch, 260cm, THSF 35x260x3) has been positioned in the left upper pulmonary vein and a 12F long delivery sheath is advanced across the atrial septum. **D**, deployment of the left atrial disc of the AFR device. **E**, the right atrial disc is deployed and the AFR-device properly positioned. **F**, after a pull maneuver, the AFR-device has been released. **G**, a transesophageal echocardiogram demonstrates good position of the AFR-device that creates a restrictive atrial left-to-right shunt (6mm). The patient’s post-procedural antithrombotic therapy consists of dual antiplatelet treatment for 6 months, i.e., acetylsalicylic acid (ASA) + clopidogrel, followed by long-term monotherapy with ASA or clopidogrel. See also *supplemental videos S1A-S1D*.

**Figure 2.** *Implantation of the Atrial Flow Regulator device gradually decrease pulmonary venous and left atrial size, indicating decompression of the left atrium in a 13 year old female with restrictive cardiomyopathy.* **A,B.** Transthoracic echocardiogram (apical 4 chamber view, 4CV) demonstrates grossly enlarged pulmonary veins and left atrium, prior (A) and two weeks (B) after the implantation of the AFR device. The device is adequate position and the endsystolic LA area has decreased from 23cm^2^ (**A**) to 17.1cm^2^ (**B**). **C,D**. Cardiac magnetic resonance imaging (4 chamber view, 4CV) two weeks after AFR-device implantation shows a strong decrease in LA size, a moderate increase in RA size, and the AFR-device in adequate position. Endsystolic Cine still frames are shown. After the AFR implantation, the left atrial endsystolic and enddiastolic volume index had decreased from 77 to 37 ml/m2 (-52%) and from 63 to 24 ml/m2 (-62%), respectively, and Qp/Qs ratio had increased from 0.94 to 1.52, indicating sufficient unloading of the LA and restrictive but significant atrial left-to-right atrial shunt.

See also *supplemental videos S2A-D*.

**References**

1. Paitazoglou C, Bergmann MW, Ozdemir R, Pfister R, Bartunek J, Kilic T, Lauten A, Schmeisser A, Zoghi M, Anker SD, Sievert H, Mahfoud F, Investigators A-P. One-year results of the first-in-man study investigating the atrial flow regulator for left atrial shunting in symptomatic heart failure patients: The prelieve study. *Eur J Heart Fail*. 2021;23:800-810

2. Rosalia L, Ozturk C, Shoar S, Fan Y, Malone G, Cheema FH, Conway C, Byrne RA, Duffy GP, Malone A, Roche ET, Hameed A. Device-based solutions to improve cardiac physiology and hemodynamics in heart failure with preserved ejection fraction. *JACC: Basic to Translational Science*. 2021;6:772-795

3. Hasenfuss G, Hayward C, Burkhoff D, Silvestry FE, McKenzie S, Gustafsson F, Malek F, Van der Heyden J, Lang I, Petrie MC, Cleland JG, Leon M, Kaye DM, investigators RL-Hs. A transcatheter intracardiac shunt device for heart failure with preserved ejection fraction (reduce lap-hf): A multicentre, open-label, single-arm, phase 1 trial. *Lancet*. 2016;387:1298-1304

4. Feldman T, Mauri L, Kahwash R, Litwin S, Ricciardi MJ, van der Harst P, Penicka M, Fail PS, Kaye DM, Petrie MC, Basuray A, Hummel SL, Forde-McLean R, Nielsen CD, Lilly S, Massaro JM, Burkhoff D, Shah SJ, Investigators RL-HI, Study C. Transcatheter interatrial shunt device for the treatment of heart failure with preserved ejection fraction (reduce lap-hf i [reduce elevated left atrial pressure in patients with heart failure]): A phase 2, randomized, sham-controlled trial. *Circulation*. 2018;137:364-375

5. Shah SJ, Feldman T, Ricciardi MJ, Kahwash R, Lilly S, Litwin S, Nielsen CD, van der Harst P, Hoendermis E, Penicka M, Bartunek J, Fail PS, Kaye DM, Walton A, Petrie MC, Walker N, Basuray A, Yakubov S, Hummel SL, Chetcuti S, Forde-McLean R, Herrmann HC, Burkhoff D, Massaro JM, Cleland JGF, Mauri L. One-year safety and clinical outcomes of a transcatheter interatrial shunt device for the treatment of heart failure with preserved ejection fraction in the reduce elevated left atrial pressure in patients with heart failure (reduce lap-hf i) trial: A randomized clinical trial. *JAMA Cardiol*. 2018;3:968-977

6. Guimaraes L, Bergeron S, Bernier M, Rodriguez-Gabella T, Del Val D, Pibarot P, Eigler N, Abraham WT, Rodes-Cabau J. Interatrial shunt with the second-generation v-wave system for patients with advanced chronic heart failure. *EuroIntervention*. 2020;15:1426-1428

7. Rajeshkumar R, Pavithran S, Sivakumar K, Vettukattil JJ. Atrial septostomy with a predefined diameter using a novel occlutech atrial flow regulator improves symptoms and cardiac index in patients with severe pulmonary arterial hypertension. *Catheter Cardiovasc Interv*. 2017;90:1145-1153

8. Sivakumar K, Rohitraj GR, Rajendran M, Thivianathan N. Study of the effect of occlutech atrial flow regulator on symptoms, hemodynamics, and echocardiographic parameters in advanced pulmonary arterial hypertension. *Pulm Circ*. 2021;11:2045894021989966

9. Muchtar E, Blauwet LA, Gertz MA. Restrictive cardiomyopathy: Genetics, pathogenesis, clinical manifestations, diagnosis, and therapy. *Circ Res*. 2017;121:819-837

10. Ware SM, Wilkinson JD, Tariq M, Schubert JA, Sridhar A, Colan SD, Shi L, Canter CE, Hsu DT, Webber SA, Dodd DA, Everitt MD, Kantor PF, Addonizio LJ, Jefferies JL, Rossano JW, Pahl E, Rusconi P, Chung WK, Lee T, Towbin JA, Lal AK, Bhatnagar S, Aronow B, Dexheimer PJ, Martin LJ, Miller EM, Sleeper LA, Razoky H, Czachor J, Lipshultz SE, Pediatric Cardiomyopathy Registry Study G. Genetic causes of cardiomyopathy in children: First results from the pediatric cardiomyopathy genes study. *J Am Heart Assoc*. 2021;10:e017731

11. Lee TM, Hsu DT, Kantor P, Towbin JA, Ware SM, Colan SD, Chung WK, Jefferies JL, Rossano JW, Castleberry CD, Addonizio LJ, Lal AK, Lamour JM, Miller EM, Thrush PT, Czachor JD, Razoky H, Hill A, Lipshultz SE. Pediatric cardiomyopathies. *Circ Res*. 2017;121:855-873

12. Webber SA, Lipshultz SE, Sleeper LA, Lu M, Wilkinson JD, Addonizio LJ, Canter CE, Colan SD, Everitt MD, Jefferies JL, Kantor PF, Lamour JM, Margossian R, Pahl E, Rusconi PG, Towbin JA, Pediatric Cardiomyopathy Registry I. Outcomes of restrictive cardiomyopathy in childhood and the influence of phenotype: A report from the pediatric cardiomyopathy registry. *Circulation*. 2012;126:1237-1244

13. Weller RJ, Weintraub R, Addonizio LJ, Chrisant MR, Gersony WM, Hsu DT. Outcome of idiopathic restrictive cardiomyopathy in children. *Am J Cardiol*. 2002;90:501-506

14. Lauder L, Pereira TV, Degenhardt MC, Ewen S, Kulenthiran S, Coats AJS, Bohm M, Anker SD, da Costa BR, Mahfoud F. Feasibility and efficacy of transcatheter interatrial shunt devices for chronic heart failure: A systematic review and meta-analysis. *Eur J Heart Fail*. 2021

15. McDonagh TA, Metra M, Adamo M, Gardner RS, Baumbach A, Bohm M, Burri H, Butler J, Celutkiene J, Chioncel O, Cleland JGF, Coats AJS, Crespo-Leiro MG, Farmakis D, Gilard M, Heymans S, Hoes AW, Jaarsma T, Jankowska EA, Lainscak M, Lam CSP, Lyon AR, McMurray JJV, Mebazaa A, Mindham R, Muneretto C, Francesco Piepoli M, Price S, Rosano GMC, Ruschitzka F, Kathrine Skibelund A, Group ESCSD. 2021 esc guidelines for the diagnosis and treatment of acute and chronic heart failure. *Eur Heart J*. 2021;42:3599-3726

16. Lewicki L, Kosmalska K, Liedtke S, Karwowski M, Siebert J, Sabiniewicz R, Kiedrzyn J, Kot A, Szolkiewicz M. Pomeranian atrial flow regulator in congestive heart failure (prolonger): Study protocol. *Cardiol J*. 2020;27:879-883
